# Feasibility of the Modified Telephone Interview for Cognitive Status (M‐TICS) in the peri‐operative environment

**DOI:** 10.1111/anae.70022

**Published:** 2025-10-14

**Authors:** Kelly J. Atkins, Lis Evered, Brendan Silbert, Joanne Robertson, Lucy Mackintosh, David Ames, David A. Scott

**Affiliations:** ^1^ Department of Anesthesiology Weill Cornell Medicine New York NY USA; ^2^ Department of Anaesthesia and Acute Pain Medicine St Vincent's Hospital Melbourne Melbourne VIC Australia; ^3^ Department of Critical Care Melbourne Medical School, University of Melbourne Melbourne VIC Australia; ^4^ The Florey Institute of Neuroscience and Mental Health Melbourne VIC Australia; ^5^ University of Melbourne Academic Unit for Psychiatry of Old Age St George's Hospital Kew Melbourne VIC Australia

**Keywords:** cognitive screening, dementia, mild cognitive impairment, peri‐operative, remote assessments

## Abstract

**Introduction:**

Peri‐operative neurocognitive disorders are common among older adults presenting for surgery and anaesthesia. Cognitive screening is recommended to identify patients at risk for adverse neurocognitive outcomes, though the most appropriate peri‐operative tool remains debated. Remote assessment methods may be advantageous, but they need robust validation. We aimed to examine the feasibility and validity of the Modified Telephone Interview for Cognitive Status (TICS‐M) among older adults and provide recommended TICS‐M scores to identify those most at risk of poor postoperative cognitive outcomes.

**Methods:**

As part of a prospective longitudinal study with 215 older adults living in the community or scheduled for elective surgery, we conducted the modified, 22‐item, 50‐point version of the TICS remotely, followed by in‐person assessments using two common cognitive screening tools: the Mini‐Mental State Examination (MMSE); Alzheimer's Disease Assessment Scale Cognition Subscale (ADAS‐Cog); and a comprehensive neuropsychological and functional assessment.

**Results:**

The TICS‐M was feasible and acceptable, with a completion rate of 86%. TICS‐M scores correlated with scores on the MMSE (r = 0.61, p < 0.001) and ADAS‐Cog (r = ‐0.55, p < 0.001) at baseline, and associations remained consistent at 12‐ and 24‐month follow‐up. After controlling for age, sex and education, baseline performance on the TICS‐M was independently associated with subsequent cognitive impairment at 12 months (OR 0.84, 95% CI 0.77–0.92, p < 0.001) and 24 months (OR 0.84, 95% CI 0.76–0.94, p = 0.001). A TICS‐M score of 32.5 was the optimal threshold to identify people with cognitive impairment (0.76, 95% CI 0.70–0.82, p < 0.001).

**Discussion:**

The TICS‐M is a feasible, valid and reliable remotely administered tool that shows utility in the peri‐operative environment. We recommend its implementation into routine clinical practice for remote pre‐operative assessment in patients aged ≥ 65 y scheduled for surgery and anaesthesia.

## Plain Language Summary

Many older people who have surgery and anaesthesia have problems with their memory or thinking. To spot who might be at risk, health professionals use tests to check how well people think and remember things. It is still being discussed which test is the best one to use before surgery. Doing these tests over the phone or remotely could be helpful, but these tests need to be carefully checked to make sure they work well. In this study, we looked at whether a test called the Modified Telephone Interview for Cognitive Status (TICS‐M) could be used for older adults. We also wanted to find out what scores on the test might show that someone is more likely to have thinking or memory problems after surgery. We worked with 215 older adults who were either living at home or waiting for planned surgery. They first did the TICS‐M test, which has 22 questions. Then, we gave them two other common thinking tests in person, called the Mini‐Mental State Examination (MMSE) and the Alzheimer's Disease Assessment Scale (ADAS‐Cog), along with other detailed memory and function tests. The TICS‐M test was found to be practical and easy for most people, with 86% of people completing it. Scores on this test matched well with scores on the other tests. These connections stayed strong when we checked again 1 year and 2 years later. After accounting for age, sex and education, we found that people's TICS‐M scores at the start could predict later memory or thinking problems. A score of 32.5 was the best cut‐off point for spotting people at risk. We found that the TICS‐M is a useful, reliable test that can be done remotely. We suggest using it as part of routine care for adults aged 65 and older before they have surgery and anaesthesia.

## Introduction

Peri‐operative neurocognitive disorders, including postoperative delirium, are the most common complications affecting patients aged > 65 y who have surgery [1, 2]. These adverse outcomes are associated with a cascade of mental and physical health challenges and a decline in independent function, alongside a substantial economic burden [3, 4]. We know that patients with pre‐existing cognitive impairment are most at risk of peri‐operative neurocognitive disorder [5, 6], yet pre‐operative cognitive screening is performed rarely [7]. Pre‐operative cognitive screening thus remains one simple and practical strategy with the potential to reduce adverse cognitive outcomes among older patients and demands further attention [8, 9, 10, 11, 12].

Clinically meaningful pre‐operative screening instruments separate people with typical cognition from those with mild or major cognitive impairments [7, 13]. To be feasible and effective in the peri‐operative setting, cognitive screening tools must also be simple and quick to perform [7, 11, 14]. Remote screening tools, including those that can be completed by telephone, virtually or online, may provide a cost‐effective advantage by reducing the need to attend healthcare settings, thereby facilitating rapid identification of people at risk of peri‐operative neurocognitive disorder, regardless of travel restrictions, geographical location, age or physical disability [15].

The modified telephone interview for cognitive status (TICS‐M) [16] is one possible screening tool and has been examined in research and clinical settings, typically with older adults [15, 17], alongside clinical populations including patients who have had a stroke [18, 19] and those with mild cognitive impairment [20] and dementia [21, 22]. Cut‐off scores vary according to the scale and scoring utilised making it difficult to compare thresholds across populations and settings. Use of the TICS‐M peri‐operatively is limited [23]. One small study showed that poorer pre‐operative performance on the TICS‐M was associated with adverse postoperative outcomes, including postoperative delirium [24]. The association of the TICS‐M with neuropsychological test performance pre‐ and postoperatively has also been evaluated, with weak to moderate correlations between tests [25]. However, follow‐up in this study was limited to 4–8 weeks postoperatively, and the authors attributed postoperative neurocognitive dysfunction based on objective neuropsychological assessments alone, omitting subjective complaints and activities of daily living. More information is needed about whether the TICS‐M is a feasible instrument for use in the peri‐operative period, whether it is associated with longer term cognitive outcomes resulting from surgery and anaesthesia, and to determine an appropriate threshold that may indicate patients at greatest risk.

The aim of this study was to examine the use of the TICS‐M in the peri‐operative setting among a subset of older Australians with a subjective memory complaint or diagnosed mild cognitive impairment. We selected older adults with a subjective memory complaint as they are most at risk of postoperative cognitive change, and thus, most likely to benefit from healthcare protocols that include pre‐operative cognitive screening [5, 26]. We compared the TICS‐M against commonly used paper and pencil tests (i.e. the Mini‐Mental State Examination (MMSE) [27] and Alzheimer's Disease Assessment Scale Cognition (ADAS‐Cog) [28]) as well as cognitive status determined by an expert panel of neurologists, geriatricians and neuropsychologists (as for the Australian Imaging and Biomarker Lifestyle study) [29]. In this study, cognitive status was based on a neuropsychological and clinical assessment, distinct from the TICS‐M assessment. We sought to determine an optimal threshold for the TICS‐M that may be used to discriminate cognitive impairment in the peri‐operative setting and extend previous studies by examining the association between pre‐operative TICS‐M performance and cognitive impairment at 12 and 24 months.

## Methods

We recruited people aged ≥ 60 y as a part of a larger longitudinal observational study evaluating cognitive change over the course of the surgical period (the AHEAD study, ACTRN12614000012673). Results of the AHEAD study are in preparation and are not reported here. Briefly, the observational AHEAD study recruited 252 surgical patients and non‐surgical community‐dwelling older adults for prospective cognitive assessment. All participants had a self‐ or informant‐reported cognitive complaint and were followed over the course of 2 years. Recruitment sources were the elective surgery waitlist at a major tertiary hospital in Melbourne, Australia, as well as referrals from a private geriatrician and community advertisements.

For the purposes of this study, we pooled responses on our outcomes of interest from both non‐surgical older adults and patients having surgery. We included people with a subjective memory complaint (self or informant‐reported) or diagnosed mild cognitive impairment and who were fluent in English. We did not study people with a history of cerebrovascular accident; traumatic brain injury; epilepsy; intellectual disability; major psychiatric illness (e.g. schizophrenia, bi‐polar disorder); those who had undergone a general anaesthetic in the preceding 12 months; and those who had a contraindication to neuropsychological testing (blindness, deafness, severe physical disability affecting hand co‐ordination). The TICS‐M was applied as part of recruitment screening (see below). The St Vincent's Hospital Melbourne Human Research Ethics Committee approved the study and oversaw the ethical compliance with the study protocol. All participants gave written informed consent.

The TICS‐M [16] is a cognitive screening tool comprising 22 questions designed to capture cognitive domains affected by mild and major neurocognitive disorders, including orientation; attention; memory; language; and verbal‐based executive functions. Possible scores range from 0 to 50, with higher scores indicating better cognitive function. We utilised an Australian adaptation of the TICS‐M and substituted questions pertaining to the name of the US President with the Australian Prime Minister and the name of the US Vice‐President with the current UK monarch. We examined raw TICS‐M scores according to standard published procedures.

All participants completed an in‐person clinical interview and cognitive assessment with a trained research assistant. This assessment informed the clinical classification of each patient. The cognitive assessment battery included the MMSE [27] and ADAS‐Cog [28], alongside neuropsychological tests of attention; processing speed; memory; language; executive functioning; and psychomotor control. We assessed mood with the Geriatric Depression Scale [30]. The clinical interviews gathered relevant demographic information such as education and vocational history; current psychosocial information such as living arrangements and social engagement; relevant psychiatric and medical history; engagement in activities of daily living; and the extent and source of memory complaints.

Potential participants were contacted via telephone or letter by trained clinical research assistants. We then screened participants to determine their eligibility and subsequently undertook the TICS‐M according to standardised test instructions. Briefly, to complete the TICS‐M, participants were asked to find a quiet space to complete the assessment, turn off any background noise (e.g. television or radios) and answer the questions without the help of anyone else present and without taking any notes. In the event participants were called at an inconvenient time (i.e. while driving, shopping, in a noisy setting), an alternative time was arranged for another phone call to complete the TICS‐M.

If eligible for the larger research study, participants elected a suitable time at which they gave their written informed consent and completed a battery of neurocognitive tests alongside self‐ and informant‐reported functional questionnaires at their home. Following completion of the comprehensive assessment, the participant information was presented to a panel of experts [29], comprised of neurologists, geriatricians, geriatric psychiatrists and neuropsychologists, who, after reaching consensus, classified participants into one of three groups: subjective memory complaint; mild cognitive impairment; or dementia. We then completed clinical and neurocognitive assessments at 12‐ and 24‐month follow‐ups and, for each time‐point, participant information was re‐presented to the expert panel and classified. Although participants completed our cognitive test battery up to three times, the TICS‐M was performed only once at the time of study enrolment.

Descriptive statistics, independent sample t‐tests, χ^2^ and Pearson correlation coefficients were used to evaluate the frequency of TICS‐M completion and the association between TICS‐M scores and participant characteristics. We used one‐way ANOVA, with Bonferroni adjusted pairwise comparisons and binomial logistic regression to examine the relationship between TICS‐M scores and cognitive classification. To establish clinically meaningful cut‐off scores, we used receiver operating characteristic (ROC) curve analyses, comparing the TICS‐M scores with the expert panel classifications that served as the gold standard. To obtain a suitable threshold for use in the peri‐operative setting, we combined participants classified by the expert panel with mild cognitive impairment or dementia into one group, who were then compared with those classified with subjective memory complaint. All analyses were performed using SPSS statistics V.26 (IBM, Armonk, NY, USA).

## Results

**Figure 1:**
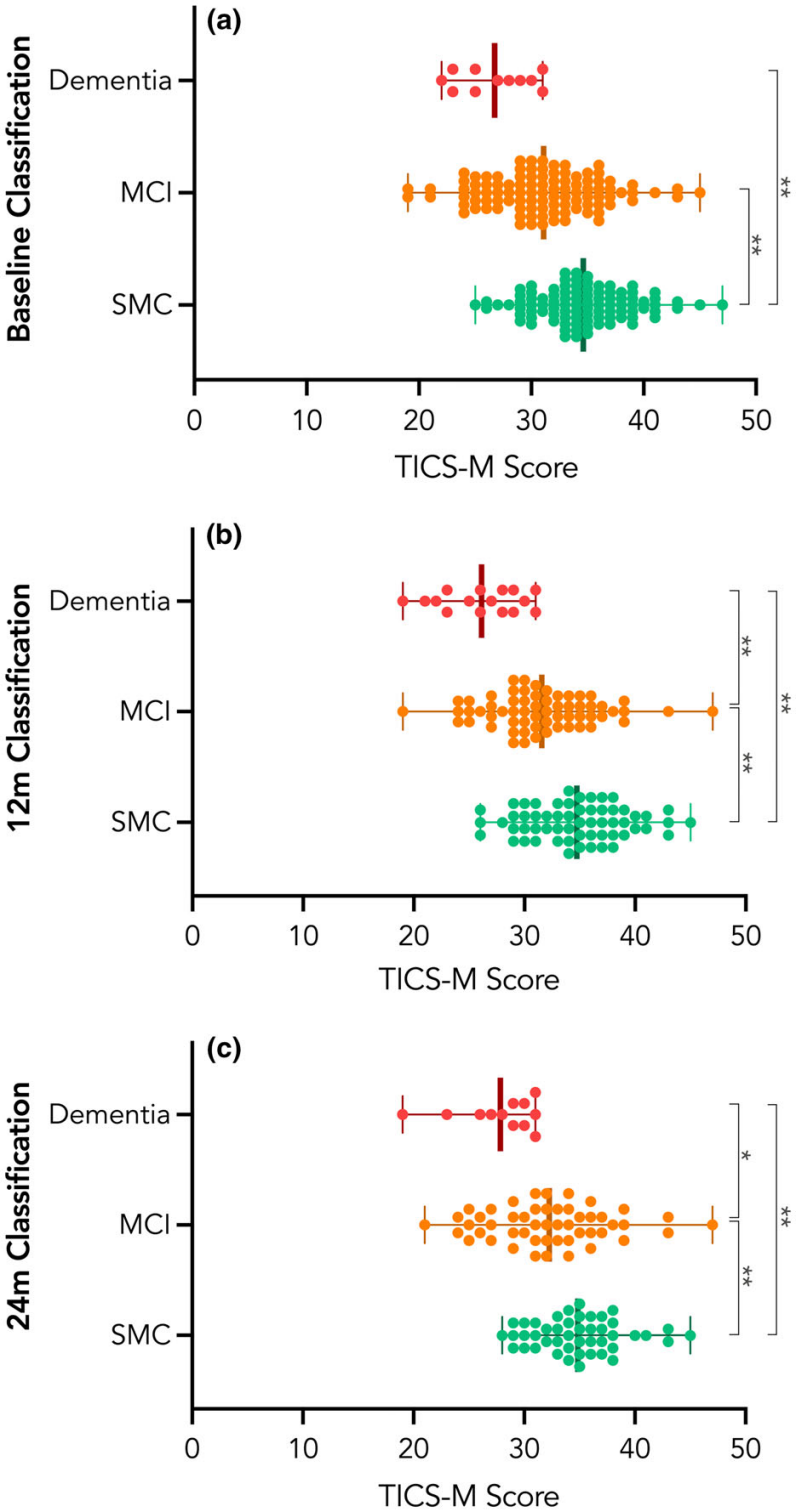
Distribution of baseline telephone interview for cognitive status‐modified (TICS‐M) scores according to cognitive classification at (a) baseline; (b) 12 months; and (c) 24 months. *Indicates difference at p < 0.05. **Indicates difference at p < 0.001. MCI, mild cognitive impairment; SMC, subjective memory complaint.

Data were collected between February 2014 and March 2022. Of the 250 participants enrolled in the AHEAD study, 215 (86%) completed the TICS‐M at baseline, alongside a neurocognitive and functional assessment. Participants who completed the TICS‐M did not differ from non‐responders with respect to age (t(247) ‐1.4, p = 0.17); education (t(231) 0.28, p = 0.78); or sex (χ^2^ 1.8, p = 0.19, n = 250). Reasons for non‐completion included both participant factors (e.g. hearing difficulties, inconvenient location or time) and staff factors (e.g. insufficient time, competing tasks). Of the 215 responders, 121 were female (56.3%) with a mean (SD) age of 74 (7.7) y (range 60–94 y). Performance on the TICS‐M was not associated with sex (t(213) 1.4, p = 0.16) or age (r(213) ‐0.12, p = 0.08). There was a moderate positive association between education and TICS‐M scores, with higher levels of education associated with better performance on the TICS‐M (r(205) 0.37, p < 0.001). At 12 months, 132 (61.4%) participants completed the follow‐up assessment and 110 (51.2%) at 24 months. The cognitive classification of participants at each time point, as determined by the expert panel, is shown in Table 1. The distribution of baseline TICS‐M scores per cognitive classification is shown in Fig. 1.

**Table 1:** Classification of participants. Data are number (proportion).

	Baseline	12 months	24 months
	n = 215	n = 132	n = 110
Subjective memory complaint	99 (46%)	55 (42%)	47 (43%)
Mild cognitive Impairment	105 (49%)	61 (46%)	51 (46%)
Dementia	11 (5%)	16 (12%)	12 (11%)

Higher TICS‐M scores were associated with better performance at baseline on the MMSE (r(212) 0.61, p < 0.001) and ADAS‐Cog (r(212) ‐0.55, p < 0.001). The association between these screening tools remained significant when they were repeated at 12 months (MMSE: r(130) 0.55, p < 0.001; ADAS‐Cog: r(130) ‐0.52, p < 0.001) and 24 months (MMSE: r(108) 0.51, p < 0.001; ADAS‐Cog: r(108) ‐0.44, p < 0.001). The performance of participants on the TICS‐M at baseline, alongside performance on the MMSE and ADAS‐Cog over time, is summarised in online Supporting Information Table S1.

The TICS‐M scores of participants classified with subjective memory complaint at baseline were significantly higher than those with mild cognitive impairment (mean difference 4.5, 95% CI 2.8–6.1, p < 0.001) and dementia (mean difference 7.9, 95% CI 4.1–11.7, p < 0.001). The TICS‐M scores did not differ statistically between the mild cognitive impairment and dementia groups (mean difference 3.4, 95% CI ‐0.3–7.2, p = 0.097). Table 1 displays the distribution of baseline TICS‐M scores with classification groups at baseline, 12 and 24 months. Participants classified with mild cognitive impairment or dementia were collapsed into one group (reflecting objective cognitive impairment). We then performed binomial logistic regression to evaluate the predictive value of the TICS‐M. We included three models, controlling for age, sex and education, with cognitive classification at baseline, 12 months and 24 months as the respective outcome variables.

In the first model, age (OR 1.1, 95% CI 1.0–1.1, p = 0.02) and TICS‐M (OR 0.8, 95% CI 0.8–0.9, p < 0.001), but not education or sex, were significantly associated with baseline cognitive classification (χ^2^(4) 51.1, p < 0.001). Similarly, at the 12‐month assessment, age (OR 1.1, 95% CI 1.0–1.2, p = 0.004) and TICS‐M (OR 0.8, 95% CI 0.8–0.9, p < 0.001) were statistically significant predictors of cognitive classification (χ^2^(4) 34.9, p < 0.001). Finally, at 24 months, the model was significant (χ^2^(4) 31.9, p < 0.001), with both age (OR 1.1, 95% CI 1.0–1.2, p = 0.003) and TICS‐M scores (OR 0.8, 95% CI 0.8–0.9, p = 0.001), as well as sex (OR 3.2, 95% CI = 1.3–8.3, p = 0.015), statistically significant predictors of cognitive classification. Education remained uninformative across all three models. The odds ratios for each of the three models are in online Supporting information Table S2.

**Figure 2:**
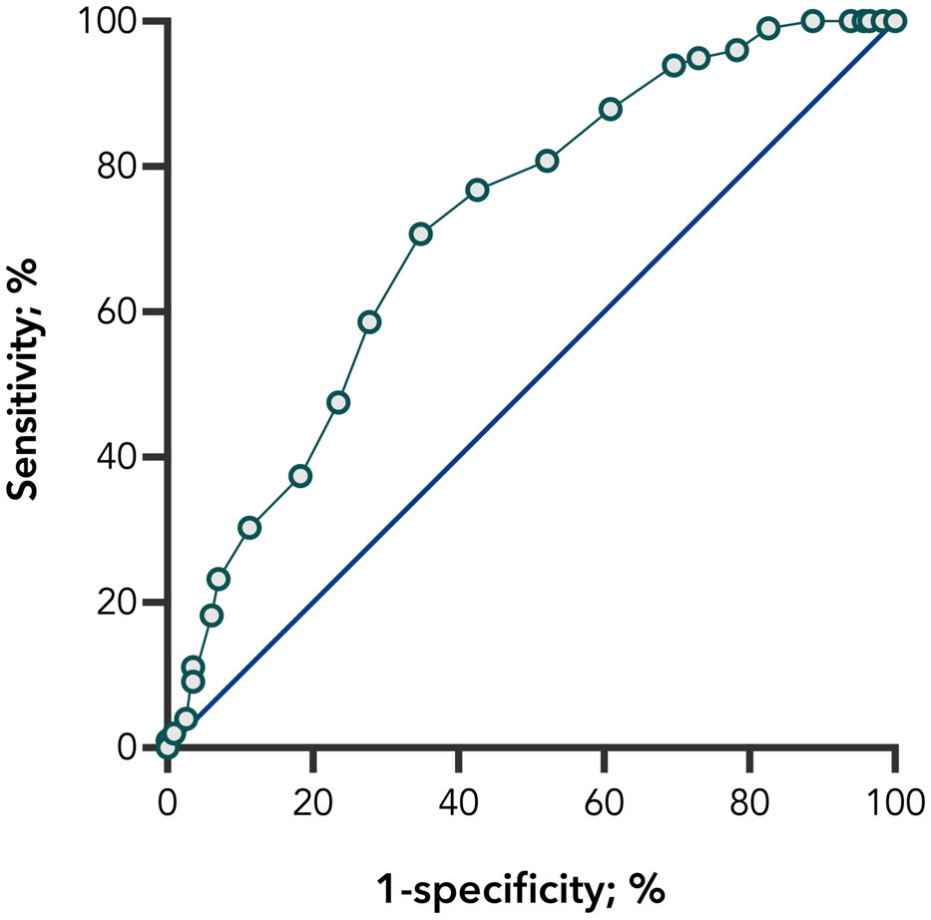
Receiver‐operator characteristic curve depicting telephone interview for cognitive status‐modified score of 32.5 as the optimal cut‐off point to identify people with cognitive impairment.

To establish TICS‐M clinical thresholds suitable for use in the peri‐operative period, we performed receiver operating characteristic (ROC) curve analyses to distinguish between those with and without objective cognitive impairment. Figure 2 shows the sensitivity and specificity of education‐adjusted TICS‐M scores using baseline cognitive classification (subjective memory complaint vs. mild cognitive impairment/dementia) as the reference criterion. The ROC curve analyses identified the optimal TICS‐M threshold to separate people with cognitive impairment from those without as 32.5 (area under the curve 0.76, 95% CI 0.70–0.82, p < 0.001).

## Discussion

We examined the feasibility of the TICS‐M, a remote screening tool, among older adults with a subjective memory complaint or mild cognitive impairment, most of whom were transitioning through a peri‐operative care setting. Overall, the TICS‐M was feasible and acceptable in our peri‐operative sample, with over 86% of participants completing the assessment. Accounting for participant factors, such as hearing impairment or unsuitable locations or times to complete remote testing, may be factored into the assessment to increase feasibility.

To establish concurrent validity, we compared the TICS‐M against commonly used paper and pencil tests (the MMSE and ADAS‐Cog) as well as cognitive classification determined by an expert panel [29]. As in previous studies, performance on the TICS‐M was mirrored by performances on the MMSE and the ADAS‐Cog, showing consistency in the sensitivity of these measures [21, 31], while retaining the advantage of remote assessment provided by the TICS‐M. Extending previous studies, we have shown the utility of the TICS‐M to identify those with objective cognitive impairment at 12 and 24 months, providing additional information beyond routine peri‐operative screening questions such as age, sex and education.

Our results indicate that the TICS‐M can equip clinicians with information about the likely cognitive trajectory of patients. In turn, this information may be used to inform the clinical priorities or alternative care pathways of patients in the intra‐operative and extended postoperative period. Considering the need to identify those most at risk of adverse neurocognitive outcomes in the peri‐operative setting, we sought to provide optimal thresholds to identify cognitive impairment among older patients. The TICS‐M thresholds we observed indicate that a score of 32.5 is the optimal threshold to identify those with cognitive impairment who are at high risk of further decline postoperatively. These thresholds are largely consistent with previously published thresholds using equivalent versions of the TICS‐M [20, 32]. Given the possible range of scores on the TICS‐M, we recommend a score of ≤ 32 in practice.

To be feasible for use in the peri‐operative environment, cognitive screening tools must account for the increasing preference to undertake pre‐operative assessments remotely over the telephone, rather than in face‐to‐face pre‐operative clinics [33]. Virtual pre‐anaesthesia evaluation has similar surgery cancellation rates, higher patient satisfaction [34] and reduced costs compared with in‐person evaluation [33]. The challenges of the COVID‐19 pandemic have further bolstered the use of remote clinics [35]. Unlike traditional screening tools such as the MMSE, the TICS‐M has the distinct advantage of a completely remote assessment protocol and is adaptive to changes in the way peri‐operative medicine is performed. Furthermore, the TICS‐M requires minimal training to carry out, is validated across cultural groups [36] and is available in more than nine languages, making it an ideal tool for pre‐operative assessments in diverse patient populations [13].

Despite best practice guidelines [10], routine pre‐operative care standards remain limited in the assessment of cognitive impairment. Population ageing statistics indicate that the number of older adults in the USA will more than double from 46 million at present to 98 million by 2060 [37]. This increase has major implications for peri‐operative medicine, with older adults accounting for more than 30% of people presenting for surgery and anaesthesia [38], many of whom will have cognitive impairment [5, 39, 40].

Our study evaluated the use of the TICS‐M as a tool to screen for cognitive impairment, but identification of those at risk is only as useful as the change in clinical care triggered by this risk [11, 38]. Clinical care settings must respond accordingly and adapt clinical pathways to assess and mitigate patient risk appropriately [38, 41]. Identifying those most at risk of adverse cognitive outcomes provides an opportunity to reduce or prevent the personal and economic costs of peri‐operative neurocognitive disorder in the short and long term [11, 26, 42]. A range of trials underway reflect this and aim to prevent peri‐operative neurocognitive disorder using peri‐operative optimisation strategies, including psychoeducation for patients and families; modifications to the hospital environment; and lifestyle strategies to reduce cardiovascular risk [41, 43, 44, 45]. Furthermore, peri‐operative care settings must develop pathways that allow communication of cognitive screening results to the multidisciplinary peri‐operative team, including surgeons, anaesthetists, neuropsychologists, physical therapists, occupational therapists and geriatricians, to thus provide a tailored and appropriate care pathway [7].

This study had some limitations. We utilised a subsample of patients enrolled in the AHEAD study. Participants were eligible for the AHEAD study if they reported a subjective memory complaint or if they had a previous diagnosis of mild cognitive impairment. An evaluation of the costs and benefits of assessing only patients who endorse a complaint or all patients presenting for surgery (over a certain age) is an important avenue of future research and aligns with questions currently under evaluation in primary care and community neurology settings [46]. Given that best practice peri‐operative guidelines recommend all older adults presenting for surgery may benefit from cognitive testing, we recommend the TICS‐M for all patients aged ≥ 65 y, but caution that our results may not generalise to a patient population without a subjective cognitive complaint. A general limitation of the TICS is the numerous scoring systems that affect the possible range of scores [17]. This hampers efforts to compare thresholds across studies and patient populations and accordingly the generalisability of our findings. Furthermore, it must be cautioned that the TICS‐M is a measure intended to screen, rather than diagnose, cognitive impairment and may be insensitive to subtle changes in cognition as well as the small, incremental changes between mild cognitive impairment and mild dementia. This limitation, combined with our sample size and that participants with dementia were largely in the mild disease stage, may have contributed to the modest differentiation observed in scores between participants with mild cognitive impairment and dementia.

In this study, we showed that the TICS‐M is a feasible, cognitive screening tool undertaken remotely, performing similar to commonly used but less flexible paper‐and‐pencil tests. Furthermore, the TICS‐M provides information about cognitive trajectory, beyond routine pre‐operative patient characteristics, to aid clinicians in determining the most appropriate clinical management and referral pathways within the acute and long‐term postoperative period.

We have shown that the TICS‐M is a suitable tool for use within the peri‐operative environment and recommend its implementation into routine clinical practice for patients aged ≥ 65 y scheduled for surgery and anaesthesia who undergo pre‐operative anaesthetic assessment. An appropriate application of this tool may help screen patients who have a reported memory complaint. We propose that a score of ≤ 32 should trigger appropriate care strategies and further evaluation to ensure adverse neurocognitive outcomes are mitigated for older patients.

## Supporting information


**Plain Language Summary**.

**Table S1.** Means (SD) of telephone interview for Cognitive Status‐Modified, Mini Mental State Examination and Alzheimer's Disease Assessment Scale – Cognitive Subscale across time.
**Table S2.** Binomial logistic regression model coefficients.
