# Supplementary material for: Feasibility of the Modified Telephone Interview for Cognitive Status (M‐TICS) in the peri‐operative environment
Source: Anaesthesia. 2025 Oct 14;81(2):240–7. doi: 10.1111/anae.70022 (PMC12803579; doi:10.1111/anae.70022)
Supplement: Supplementary file 1 — Plain Language Summary. [file ANAE-81-240-s002.docx]

**Plain Language Summary**

Many older people who have surgery and anaesthesia can have problems with their memory or thinking. To spot who might be at risk, doctors use tests to check how well people think and remember things. It is still being discussed which test is the best one to use before surgery. Doing these tests over the phone or remotely could be helpful, but they need to be carefully checked to make sure they work well. In this study, we looked at whether a test called the modified Telephone Interview for Cognitive Status (TICS-M) could be used for older adults. We also wanted to find out what scores on the test might show that someone is more likely to have thinking or memory problems after surgery. We worked with 215 older adults who were either living at home or waiting for planned surgery. They first did the TICS-M test, which has 22 questions. Then, we gave them two other common thinking tests in person, called the Mini-Mental State Examination (MMSE) and the Alzheimer’s Disease Assessment Scale (ADAS-Cog), along with other detailed memory and function tests. The TICS-M test was found to be practical and easy for most people, with 86% of them completing it. Scores on this test matched well with scores on the other tests. These connections stayed strong when we checked again one year and two years later. After accounting for age, sex and education, we found that people’s TICS-M scores at the start could predict later memory or thinking problems. A score of 32.5 was the best cut-off point for spotting people at risk. We found that the TICS-M is a useful, reliable test that can be done remotely. We suggest using it as part of routine care for adults aged 65 and older before they have surgery and anaesthesia.
