# Supplementary material for: Feasibility of the Modified Telephone Interview for Cognitive Status (M‐TICS) in the peri‐operative environment
Source: Anaesthesia. 2025 Oct 14;81(2):240–7. doi: 10.1111/anae.70022 (PMC12803579; doi:10.1111/anae.70022)
Supplement: Supplementary file 2 — Table S1. Means (SD) of telephone interview for Cognitive Status‐Modified, Mini Mental State Examination and Alzheimer's Disease Assessment Scale – Cognitive Subscale across time. Table S2. Binomial logistic regression model coefficients. [file ANAE-81-240-s001.docx]

**Table S1 – Mean (SD) of TICS-M, MMSE, and ADAS-Cog across time**

|  | **Baseline** | **12-Months** | **24-Months** |
| --- | --- | --- | --- |
| TICS-M  [range] | 31.3 (5.5)  [17-45] | - | - |
| MMSE  [range] | 26.7 (2.2)  [18-30] | 26.3 (2.6)  [15-30] | 26.3 (2.8)  [17-30] |
| ADAS-Cog  [range] | 9.8 (4.7)  [1-25] | 10.4 (5.8)  [2-38] | 9.9 (4.8)  [1-23] |

TICS-M: Telephone Interview for Cognitive Status-Modified; MMSE, Mini Mental State Examination; ADAS-Cog: Alzheimer’s Disease Assessment Scale – Cognitive Subscale. The TICS-M has a possible range of 0 – 50. The possible range of scores on the MMSE is 0 – 30. The possible range of scores on the ADAS-COG is 0 – 70.

**Table S2 – Binomial logistic regression model coefficients**

| Baseline Cognitive Classification | | | | |
| --- | --- | --- | --- | --- |
| Predictor | Wald statistic | p- value | Odds Ratio | 95%CI |
| Gender | .40 | .53 | 1.22 | .65 – 2.29 |
| Education | 1.21 | .27 | .94 | .84 – 1.05 |
| Age | 5.33 | .021 | 1.05 | 1.01 – 1.10 |
| TICS-M | 24.77 | <0.001 | .83 | .78 – .90 |
| 12-Month Cognitive Classification | | | | |
| Predictor | Wald statistic | p- value | Odds Ratio | 95%CI |
| Gender | 3.10 | 0.78 | 2.10 | .92 – 4.81 |
| Education | .09 | .76 | .98 | .84 – 1.34 |
| Age | 8.26 | .004 | 1.09 | 1.03 – 1.16 |
| TICS-M | 13.78 | <.001 | .84 | .77 – .92 |
| 24-Month Cognitive Classification | | | | |
| Predictor | Wald statistic | p- value | Odds Ratio | 95%CI |
| Gender | 5.88 | .02 | 3.22 | 1.25 – 8.30 |
| Education | .88 | .35 | .92 | .77 – 1.10 |
| Age | 8.73 | .003 | 1.10 | 1.03 – 1.18 |
| TICS-M | 10.18 | <.001 | .84 | .76 – .94 |

TICS-M, Telephone Interview for Cognitive Status – Modified.
